# Analysis of Stomach and Gut Microbiomes of the Eastern Oyster (*Crassostrea virginica*) from Coastal Louisiana, USA

**DOI:** 10.1371/journal.pone.0051475

**Published:** 2012-12-12

**Authors:** Gary M. King, Craig Judd, Cheryl R. Kuske, Conor Smith

**Affiliations:** 1 Department of Biological Sciences, Louisiana State University, Baton Rouge, Louisiana, United States of America; 2 Bioscience Division, MS 888, Los Alamos National Laboratory, Los Alamos, New Mexico, United States of America; University of Waterloo, Canada

## Abstract

We used high throughput pyrosequencing to characterize stomach and gut content microbiomes of *Crassostrea virginica*, the Easter oyster, obtained from two sites, one in Barataria Bay (Hackberry Bay) and the other in Terrebonne Bay (Lake Caillou), Louisiana, USA. Stomach microbiomes in oysters from Hackberry Bay were overwhelmingly dominated by Mollicutes most closely related to *Mycoplasma*; a more rich community dominated by Planctomyctes occurred in Lake Caillou oyster stomachs. Gut communities for oysters from both sites differed from stomach communities, and harbored a relatively diverse assemblage of phylotypes. Phylotypes most closely related to *Shewanella* and a Chloroflexi strain dominated the Lake Caillou and Hackberry Bay gut microbiota, respectively. While many members of the stomach and gut microbiomes appeared to be transients or opportunists, a putative core microbiome was identified based on phylotypes that occurred in all stomach or gut samples only. The putative core stomach microbiome comprised 5 OTUs in 3 phyla, while the putative core gut microbiome contained 44 OTUs in 12 phyla. These results collectively revealed novel microbial communities within the oyster digestive system, the functions of the oyster microbiome are largely unknown. A comparison of microbiomes from Louisiana oysters with bacterial communities reported for other marine invertebrates and fish indicated that molluscan microbiomes were more similar to each other than to microbiomes of polychaetes, decapods and fish.

## Introduction

The Eastern oyster, *Crassostrea virginica*, is well known for its commercial value and importance as an “ecosystem engineer” [1]–[3]. Volumes have been written about its biology and ecology, including interactions with bacteria and other microbes. Much of this literature has emphasized diseases [4], [5] and the presence of human pathogens, especially *Vibrio parahaemolyticus* and *V*. *vulnificus* [6]–[9].

Many studies have addressed other aspects of oyster-bacteria interactions. *Cristispira* has been identified as a symbiont associated with the crystalline style, a molluscan digestive structure [10]. *Stappia* (now *Labrenzia*) has been isolated from *C*. *gigas* and *C*. *virginica*, and in the latter implicated as an antagonist for the etiological agent of Juvenile Oyster Disease [11]. Culture-dependent studies have characterized *Vibrio* and other genera associated with bulk animals and specific tissues [6]–[8], [12], [13] including identification of “indigenous” bacteria in *C. gigas* haemolymph [14], [15]. Such studies have also shown that an Eastern Mediterranean oil spill did not affect oyster-associated bacteria [16]. Culture-independent studies have documented patterns of diversity among different populations and tissues, compared hatchery-raised and wild animals, and identified the ε-Proteobacterium, *Arcobacter*, as a major contributor to the microbial community of the Chilean oyster, *Tiostrea chiliensis* [17].

Despite the pathogen-associated and fingerprinting studies summarized above, and the potential importance of bacteria for oyster nutrient acquisition, surprisingly little information exists on oyster stomach and gut microbiome diversity. Although pH values of stomach and gut tissues are similar, and particle transit times relatively short (about 1–2 h) during active feeding [18], it is unclear whether characteristic communities exist in the contents of these tissues; it is equally uncertain how microbiomes might vary within a population or across populations. To address these questions, we obtained two sets of triplicate animals, one set each from Hackberry Bay and Lake Caillou in coastal Louisiana during summer, 2010. These two geographically distinct sites (Barataria Bay and Terrebonne Bay, respectively) represent economically important sources of oysters, and experience similar salinity regimes and variability [19]. We separately collected stomach and gut contents, and sequenced PCR-amplified 16S rRNA genes using a pyrosequencing platform (Roche Diagnostics 454 Titanium). The results revealed substantial differentiation between stomach and gut microbiomes of animals from one site (Lake Caillou), but somewhat less differentiation for the second site (Hackberry Bay). Notably, Mollicutes accounted for >80% of all bacterial sequences in the stomach microbiomes of Lake Caillou oysters, but <10% of Hackberry Bay oysters. Stomach OTUs also included Actinobacteria, Chloroflexi, Firmicutes, Planctomycetes, Proteobacteria, and Spartobacteria. Chloroflexi, Mollicutes, Planctomycetes and Spartobacteria might comprise a putative core stomach microbiome, while Chloroflexi, Firmicutes, α-Proteobacteria and Verrucomicrobia might contribute to a putative core gut microbiome.

## Materials and Methods

### Sample Collection

Oysters were collected on August 4, 2010 from Hackberry Bay, a small bay adjoining Barataria Bay, Louisiana, USA. This site was unaffected by oil from the Deepwater Horizon oil spill [20]. Triplicate oysters were held on ice (<6 h) for initial processing at the Louisiana Sea Grant Oyster Hatchery, Grand Isle, LA, USA. The external valves were thoroughly cleaned to remove surface contamination, and then carefully opened leaving the animal intact. Stomach contents of individual animals were sampled using 23-gauge needles and 1-cm^3^ syringes, yielding about 0.2 cm^3^ of fluid, which was transferred to sterile 1.5 cm^3^ microfuge tubes. Gut contents were obtained by locating the intestine of individual animals and then carefully extruding hindgut material from the anus into sterile 1.5 cm^3^ microfuge tubes. Stomach and gut contents were transported on ice to a laboratory at Louisiana State University (LSU) where DNA was extracted using a MoBio PowerMax soil extraction kit (MoBio Laboratories, Inc., Carlsbad, CA) following the manufacturer’s instructions with the addition of a freeze (at −80°C, 10 min)/thaw (at 60°C, 5 min) cycle repeated three times. A second set of oysters collected on September 1, 2010 from Caillou Bay (Caillou Lake), Louisiana, USA were processed similarly with the exception that animals were transported on ice to the LSU laboratory prior to sampling stomach and gut contents. This site was also unaffected by the Deepwater Horizon oil spill. Sampling permits were not required for either site.

### DNA Analysis

DNA extracts from all samples were amplified by PCR with Platinum high-fidelity DNA polymerase (Life Technologies Corp, La Jolla, CA) in 25 µl reactions using standard protocols with the exception of a 68°C extension temperature, and primers 515f and 806r modified with barcodes and adaptors for sequencing using the Roche 454 pyrosequencing platform with titanium chemistry [21]. Each reaction mixture contained 11.5 µl water, 2.5 µl 10X high-fidelity buffer (Life Technologies Corp, La Jolla, CA), 0.75 µl of 100 mM dNTPs, 1 µl MgSO_4_, 5 µl of 0.5 mg ml^−1^ BSA, 1.5 µl for each of 515f and 806r primers, 0.2 µl high-fidelity DNA polymerase (Life Technologies Corp, La Jolla, CA), and 1 µl of extracted DNA. Reaction mixtures were denatured for 3 min at 94°C, followed by 26 cycles of 94°C for 1 min, 1 min at 54°C, and 2 min at 68°C, with a 10 min extension step at 68°C after the cycles were complete. Triplicate reactions for each sample were pooled, and then a final mixture was prepared for sequencing by adding amplicons from each sample in equal masses. Pyrosequencing was conducted by the Los Alamos National Laboratory sequencing facility, resulting in a total of 237,842 raw reads with an average length of 295 bp. Sequences have been submitted to the MG-RAST server as 4501864.3-4501873.3 (http://metagenomics.anl.gov/linkin.cgi?project=1994).

### Sequence Analysis

Raw sequences with quality scores were processed using three pipelines. PANGEA [22] was used to compare the phylogenetic composition of samples for which OTUs were classified using MEGABLAST with a reference database containing 170,273 full-length 16S rRNA gene sequences from Bacteria and Archaea isolates. Raw reads were screened using default values (average quality score, 20; minimum length, 100 bp) [22]. Reads were binned based on barcodes, which were trimmed prior to MEGABLAST. Sequences were assigned to domain/phylum, class/order/family and genus and species levels, respectively, using similarity threshold values of 0.8, 0.9, 0.95 and 0.99 for [22]. Sequences not classified by MEGABLAST were clustered into OTUs based on the same similarity thresholds. PANGEA also created a second analysis in which all samples consisted of an equal number of reads; these normalized sample datasets were constructed using sequences randomly chosen without replacement from the original screened sample files. The compositions of stomach and gut samples were compared using principal components analysis after eliminating singletons (sequences represented only once in the full dataset), and after removing cyanobacterial and eukaryotic sequences (chloroplast and mitochondrial 16S rRNA from algal cells in stomachs and guts). Of the remaining sequences identified at a phylum level or lower, representative sequences for OTUs accounting for ≥0.1% of the total were curated manually using MEGABLAST in GenBank. Any sequences misidentified by PANGEA were reclassified as necessary.

The CloVR pipeline [23] was used with its default settings (e.g., average quality score, 25; minimum length, 100 bp) to create analyses based on taxonomic affiliations (i.e., sample composition) and sequence phylogeny. For this purpose, CloVR used a hybrid pipeline consisting of Mothur sub-routines that classified sequences with the RDP database, and QIIME sub-routines for various statistical analyses. After removing cyanobacterial and eukaryotic sequences, OTUs classified by CloVR that accounted for ≥0.1% of the total remaining reads were subjected to manual curation as above. Aligned representative sequences for classified, curated OTUs were then used for a Fast UniFrac analysis (http://bmf2.colorado.edu/fastunifrac/) based on a neighbor-joining tree as input.

The Mothur pipeline [24] was used with more stringent values than the other platforms for sequence trimming (i.e., a moving window of 50 bp with an average quality score of 35; minimum length, 100 bp). The “classify” function of the Mothur pipeline was used to identify sample composition for OTUs representing ≥0.1% of the database after removing cyanobacterial and eukaryotic sequences. The remaining sequences were curated as above. These curated sequences plus the minor OTUs excluding singletons were used to generate diversity indices for the samples independent of taxonomic identifications (e.g., Shannon, inverse Simpson’s and evenness indices).

## Results

The pre-processing routines of the three pipelines employed in this study resulted in markedly different sequence numbers for analysis (Table S1). PANGEA yielded the greatest read number (199,592), and Mothur yielded the least (45,626). Sequences most closely related to cyanobacteria and eukaryotes (chloroplasts and mitochondrial 16S rRNA genes) dominated the trimmed data sets (>70%) irrespective of their size (Table S1). These sequences were eliminated from further analyses. Singleton sequences represented from 0.5% (CloVR) to 5.9% (PANGEA) of the data sets after pre-processing; these sequences were also eliminated to minimize impacts of sequencing error. Chimeric sequences were not identified in by PANGEA, but appeared to constitute only a small fraction (<0.2%) of the total sequence set (Table S1) based on results from CloVR and Mothur.

**Figure 1:**
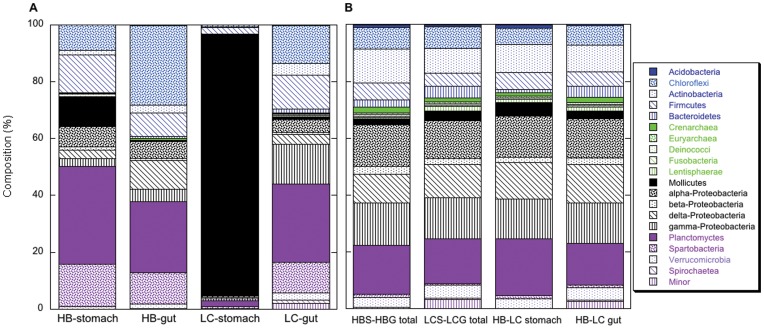
Composition of oyster stomach and gut microbiomes. A. Phylogenetic composition (phyla and classes) of Hackberry Bay (HB) and Lake Caillou (LC) stomach and gut microbiomes based on frequencies of occurrence within the set of all classified sequences. B. As for A, but composition is based on the unweighted abundance of phyla and classes with the 284 classified OTUs (all results from CloVR analysis).

Several patterns appeared consistently. The relative abundance of OTUs as a percentage of the number of sequences analyzed showed that Lake Caillou oyster stomach and gut microbiome compositions differed substantially (Fig. 1a; Tables 1, 2). A small number of Mollicute OTUs dominated the former, while Chloroflexi (mostly Caldilineae), Firmicutes, γ-Proteobacteria and Verrucomicrobia (Spartobacteria) dominated the later. All three pipelines also revealed differences between Hackberry Bay oyster stomach and gut microbiomes (Fig. 1a; Tables 1, 2), but the differences were less pronounced than those for Lake Caillou oysters. Differences among the Hackberry Bay stomach and gut microbiomes resulted primarily from modest changes in multiple lineages (e.g., Chloroflexi, Firmicutes, α-Proteobacteria, δ-Proteobacteria, Planctomycetes and Spartobacteria). In addition, each of the pipelines revealed distinct differences between the microbiomes of the two populations from Hackberry Bay and Lake Caillou. The most notable differences occurred between the two sets of stomach microbiomes, with somewhat less differentiation between the gut microbiomes (Fig. 1a, Tables 1, 2).

**Table 1:** Stomach microbiome compositions of *C. virginica* from Hackberry Bay and Lake Caillou, Louisiana determined by three pipelines.

	HB-S			LC-S		
Phylum/Class	PANGEA	CloVR	Mothur	PANGEA	CloVR	Mothur
Actinobacteria	2.65±2.12	**1.52±0.25**	1.29±0.14	0.15±0.04	**0.36±0.18**	0.30±0.17
Bacteroidetes	0.09±0.04	0.15±0.08	–	0.04±0.02	0.07±0.07	0.04±0.04
Total Firmicutes	11.41	8.86	11.37	1.15	1.90	2.09
Bacilli	9.62±6.80	**8.02±1.19**	9.17±1.81	1.11±0.52	**1.84±0.35**	1.96±0.45
Clostridia	1.79±1.21	0.84±0.42	2.20±0.92	0.04±0.02	0.06±0.02	0.13±0.07
Mollicutes	5.37±3.05	**8.37±7.83**	9.11±7.97	79.07±7.47	**86.09±4.34**	88.12±3.76
Total Proteobacteria	5.11	10.35	11.97	1.91	1.53	1.71
alpha-Proteobacteria	1.79±0.66	5.83±3.44	6.04±4.32	1.30±0.56	0.40±0.14	0.40±0.15
beta-Proteobacteria	1.19±0.67	1.23±0.68	1.03±0.84	0.06±0.03	0.09±0.02	0.05±0.01
delta-Proteobacteria	1.14±0.32	1.81±0.73	2.58±0.35	0.17±0.03	0.28±0.14	0.53±0.22
gamma-Proteobacteria	0.99±0.31	1.48±0.36	2.32±0.44	0.38±0.09	0.76±0.38	0.73±0.35
Total Chloroflexi	8.03	8.02	7.74	0.23	0.68	0.54
Anaerolineae	0.11±0.04	–	–	0.01±0.00	–	0.04±0.02
Caldilineae	0.12±0.10	**8.02±1.34**	7.74±0.56	0.02±0.02	**0.68±0.29**	0.50±0.19
Planctomycetes	23.29±18.08	**32.74±9.51**	29.31±9.27	1.65±0.43	**2.07±0.52**	1.76±0.42
Total Verrucomicrobia	3.49	12.53	13.56	0.43	1.12	0.97
Spartobacteria	3.15±1.01	–	12.78±1.70	0.32±0.06	–	0.87±0.33
Verrucomicrobiae	0.33±0.11	**12.53±1.37**	0.65±0.34	0.11±0.03	**1.12±0.45**	0.06±0.03
Crenarchaea	0.15±0.14	–	0.13±0.13	0.02±0.02	–	0.02±0.02
Euryarchaea	–	0.21±0.11	0.39±0.00	–	0.08±0.04	–
Deinococci	0.11±0.03	0.52±0.20	0.65±0.34	0.03±0.02	0.04±0.02	0.09±0.07
Fusobacteria	0.23±0.23	–	–	–	–	–
Unclassified	39.65±10.54	**16.58±2.18**	14.49±3.97	15.30±7.70	**5.97±2.53**	4.25±2.07
Total	99.58	99.85	99.87	99.96	99.92	99.84

Values for each class or phylum are percentages of the total non-eukaryotic sequences for triplicate samples with standard errors for taxa represented at more than 0.1% in at least one pipeline. These taxa account for >99.5% of all bacterial sequences. Rare occurrences were noted for Chlamydiae, Chlorobi, Dictoglomia, Lentisphaerae, Spirochaeta, Synergistes and Thermomicrobia. Bold indicates that comparisons of HB and LC for the CloVR pipeline are statistically different at p<0.05.

**Table 2:** Hackberry Bay and Lake Caillou oyster gut microbiome compositions and values as in Table 1.

	HB-G			LC-G		
Phylum/Class	PANGEA	CloVR	Mothur	PANGEA	CloVR	Mothur
Acidobacteria	0.03±0.02	0.32±0.08	0.21±0.03	0.02±0.01	0.40±0.10^#^	0.06±0.03
Actinobacteria	1.00±0.69	2.32±0.68	2.27±0.46	2.51±0.55	2.96±0.57^#^	3.37±0.81
Bacteroidetes	0.29±0.13	0.20±0.10	0.60±0.04	0.62±0.51	0.69±0.47	1.01±0.71
Chlamydiae	0.07±0.03	0.19±0.15	–	0.19±0.02	0.13±0.03^#^	0.08±0.08
Ktedonobacteria	–	–	–	–	–	0.12±0.06
Lentisphaerae	–	–	0.06±0.06	–	–	0.36±0.09
Spirochaeta	–	–	0.20±0.20	0.38±0.27	0.66±0.50	1.17±0.76
Synergistes	–	–	–	–	–	0.93±0.79
Total Firmicutes*	3.16	3.17	7.11	4.78	6.40	9.93
Bacilli	1.82±1.48	1.74±1.11^#^	1.71±0.28	4.25±2.64	6.09±4.04	7.51±0.45
Clostridia	1.34±0.65	**1.43±0.18**	5.40±0.17	0.53±0.06	**0.31±0.08^#^**	0.86±0.09
Mollicutes	5.04±4.80	**0.03±0.03**	0.07±0.07	1.48±0.43	**1.57±0.44^#^**	0.75±0.44
Total Proteobacteria	11.26	14.44	17.86	21.12	23.57	19.10
alpha-Proteobacteria	3.57±0.88	**5.36±0.58**	4.99±0.70	2.64±0.49	**2.86±0.22^#^**	3.74±0.09
beta-Proteobacteria	0.23±0.07	0.84±0.04	0.71±0.15	0.73±0.23	0.86±0.20^#^	0.60±0.16
delta-Proteobacteria	2.74±0.51	**6.45±0.57^#^**	8.52±0.64	2.46±0.33	**1.55±0.63**	2.95±0.82
gamma-Proteobacteria	4.60±3.01	1.79±0.33	3.64±0.77	15.20±9.36	17.97±11.03	11.41±6.43
epsilon-Proteobacteria	0.02±0.02	–	–	0.09±0.07	0.33±0.17	0.40±0.21
Total Chloroflexi*	5.90	22.13	23.96	6.68	9.79	10.92
Anaerolineae	0.06±0.02	–	0.38±0.15	0.57±0.29	–	0.60±0.16
Caldilineae	0.06±0.03	**22.13±1.90^#^**	23.58±1.77	0.22±0.13	**9.79±1.81^#^**	10.26±1.94
Planctomycetes	7.19±2.28	22.48±0.29	21.39±0.83	15.42±1.76	19.98±2.35^#^	22.77±2.67
Total Verrucomicrobia	5.55	9.31	10.80	7.22	9.47	10.96
Spartobacteria	4.93±1.35	–	9.34±1.37	5.98±1.23	–	8.93±1.58
Verrucomicrobiae	0.62±0.17	9.31±0.82	0.59±0.34	1.24±0.12	9.47±1.98^#^	0.80±0.40
Crenarchaea	0.01±0.01	–	0.44±0.12	0.05±0.04	–	0.08±0.08
Euryarchaea	–	0.03±0.03	0.20±0.20	–	0.14±0.05	0.21±0.02
Deinococci	0.05±0.05	0.03±0.03	–	0.09±0.06	0.11±0.07	0.21±0.16
Fusobacteria	0.02±0.01	0.16±0.12	0.20±0.20	0.29±0.11	0.30±0.09^#^	0.31±0.17
Unclassified	60.43±3.34	25.18±1.21^#^	14.69±0.12	38.89±8.07	23.82±6.37	17.52±2.42
Total	100.00	99.99	99.99	99.64	99.99	99.86

Taxa shown account for >99.6% of all bacterial sequences. Rare occurrences were noted for Chlorobi, Dictoglomia, Desferribacters, Nitrospirae, Thermobaculum, TM7 and WS3. Bold indicates statistical significance as in Table 1. Asterisks indicate that totals include unclassified members of a phylum. A superscript # indicates a statistically significant difference (p<0.05) between stomach and gut compositions using the CloVR pipeline.

In spite of many similarities, PANGEA, CloVR and Mothur output differed in important respects. Relative to CloVR and Mothur, PANGEA identified fewer Proteobacteria, Mollicutes and Verrucomicrobia in Hackberry Bay oyster stomach microbiomes, and fewer Actinobacteria, Chloroflexi, Planctomycetes, and Verrucomicrobia in gut microbiomes. PANGEA also consistently recorded a larger percentage of “unclassified” sequences than did CloVR or Mothur; PANGEA did not identify 60% of the Hackberry Bay oyster stomach sequences beyond the domain level (Tables 1, 2).

Differences were also observed in the taxonomic affiliations of the most abundant OTUs (Table 3). PANGEA, CloVR and Mothur all reported Planctomycetes as one of two equally most abundant OTUs in Hackberry Bay oyster stomach microbiomes, but the specific affiliations within the Planctomycetes differed. The affiliations of the second OTU also differed, including a Firmicute (PANGEA), Spartobacteria (CloVR) and Mollicute (Mothur). In addition, PANGEA reported a sequence related to *Mycoplasma mobile* as the most abundant OTU for Hackberry Bay oyster gut microbiomes, while the other pipelines reported a Chloroflexi strain (Table 3). In contrast, the three pipelines showed much closer agreement for Lake Caillou samples: all found that an OTU closely related to *M*. *mobile* was most abundant in stomach microbiomes, and an OTU closely related to a *Shewanella* sp. was most abundant in gut microbiomes. The two *Shewanella* isolates reported, MOLA 59 (PANGEA) and THt8-1 (CloVR and Mothur), were identical over the nucleotide positions analyzed. However, *Shewanella* sp. THt8-1 and *Shewanella* sp. MOLA 59 were isolated from terrestrial plant and marine sources, respectively.

**Table 3:** Taxonomic affiliation of the most abundant OTUs (evolutionary distance = 0.03) in *Crassostrea virginica* stomach (S) and gut (G) microbiomes for Hackberry Bay (HB) and Lake Caillou (LC) as determined by three different sequence analysis pipelines.

Group	PANGEA	CloVR	Mothur
HBS	*Pirellula* sp. Schlesner 139 (96%)	Planctomycete MS1316 (93%)	Planctomycete str. 116 (90%)
	*Falklamia* sp. H119 (89%)	Bacterium Ellin507 (92%)	*Mycoplasma mobile* (92%)
HBG	*M*. *mobile* (93%)	Chloroflexi str. ET-1 (93%)	Chloroflexi str. ET-1 (93%)
LCS	*M*. *mobile* (93%)	*M*. *mobile* (93%)	*M*. *mobile* (93%)
LCG	*Shewanella* sp. MOLA 59 (99%)	*Shewanella* sp. THt8-1 (99%)	*Shewanella* sp. THt8-1 (99%)

Bacterium Ellin507 belongs to the Spartobacteria and *Falklamia* sp. H119 to the Firmicutes (Bacilli).

Analyses of the composition (phyla and classes) of the 284 classified OTUs (Fig. 1b) revealed patterns that diverged somewhat from those based on relative abundance of phyla and classes among all sequences (Fig. 1a). First, differences between stomach and gut microbiomes within a site and across sites based on OTU composition were less pronounced than those based on frequencies of occurrence (Fig. 1a vs. 1b). This was evident for major (e.g., Chloroflexi, Firmicutes, γ-Proteobacteria, δ-Proteobacteria and Planctomyces) and minor (e.g., Archaea, β-Proteobacteria, and Spartobacteria) contributors to OTU composition (Fig. 1b). Second, the percentage contribution of some phyla and classes to the classified OTUs was substantially overrepresented relative to their abundance in the sequence data set, while other phyla and classes were substantially underrepresented (Fig. 1a, b). Mollicutes were greatly overrepresented in Hackberry Bay and Lake Caillou stomach microbiomes, but underrepresented in gut microbiomes. Chloroflexi and Planctomyces were also overrepresented in Lake Caillou oyster gut and Hackberry Bay oyster stomach and gut microbiomes, while α-, and β-Proteobacteria were underrepresented in all microbiomes (Fig. 1a, b).

**Figure 2:**
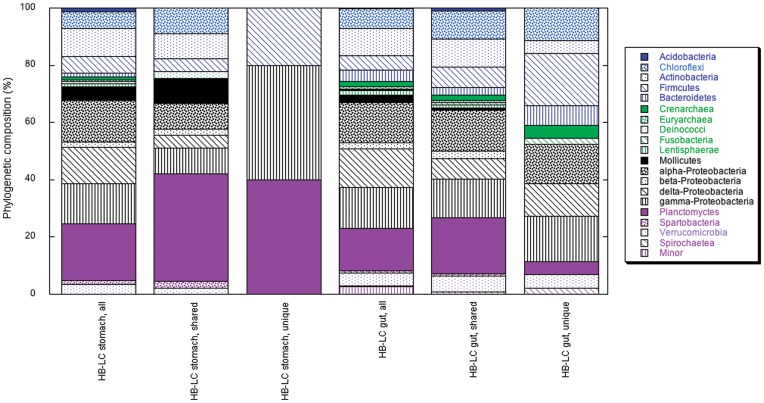
Phylogenetic composition (phyla and classes) of all OTUs found in pooled Hackberry Bay (HB) and Lake Caillou (LC) stomach or gut microbiomes; OTUs found in (shared among) all stomach replicates or all gut replicates for both sites (SHR-S and SHR-G, respectively); OTUs found exclusively in all stomach or gut replicates (SHRU-S and SHRU-G, respectively). See text for further details.

An analogous pattern was observed when the phylogenetic composition of all OTUs that occurred in pooled Hackberry Bay and Lake Caillou stomach microbiomes was compared with the composition of OTUs that occurred in or were shared (SHR-S) across both sites. In particular, Chloroflexi, Mollicutes, Planctomyces and Spartobacteria were overrepresented among the SHR-S OTUs (Fig. 2). Similarly, a comparison of OTUs occurring in pooled Hackberry Bay and Lake Caillou gut microbiomes with the shared gut OTUs (SHR-G) showed that Chloroflexi, Firmicutes, α-Proteobacteria, Planctomyces and Verrucomicrobia were overrepresented (Fig. 2). The number of SHR-S OTUs (44) was much smaller than the number of SHR-G OTUs (112), the latter of which accounted for almost 40% of all classified OTUs, and an even larger percentage of those found in the gut microbiomes (Table 4).

**Table 4:** Numbers of OTUs observed for Hackberry Bay (HB) and Lake Caillou (LC) stomach and gut (S, G) microbiomes, and numbers of OTUs shared among samples based on sequences classified through the Mothur pipeline.

	HBS	HBG	LCS	LCG
OTU total	138	243	172	304
Classified OTUs	95	166	121	214
% Unclassified	31.2	31.7	29.7	29.6
	**HBS-HBG**	**LCS-LCG**	**HBS-LCS**	**HBG-LCG**
Total Shared	64	95	44	112
% of Total Classified^a^	22.5	33.5	15.5	39.4

a Total classified refers to the sum for all gut and stomach samples (284).

Among all samples, 401 bacterial OTUs were observed at an evolutionary distance of 0.05–0.10, of which 284 were classified to at least the level of class. Values for HBS-HBG, LCS-LCG, HBS-LCS, and HBG-LCG represent classified OTUs shared between HB stomach and gut microbiomes, Lake Caillou stomach and gut microbiomes, HB and LC stomach microbiomes, and HB and LC gut microbiomes, respectively.

OTUs that occurred uniquely in stomach or gut microbiomes of both Hackberry Bay and Lake Caillou oyster populations (SHRU-S, SHRU-G) represented another distinct sub-group. The SHRU-S microbiome was represented by just 5 of the 44 SHR-S OTUs in only 3 phyla/classes, and accounted for only 2.1% of the 284 total OTUs identified in the collective stomach and gut microbiomes (Table 4). In contrast, the SHRU-G microbiome were represented by 44 of the 112 SHR-G OTUs in 12 phyla/classes, and accounted for 15.5% of all identified OTUs (Table 4). The composition of SHR-S and SHRU-S microbiome OTUs differed markedly, while differences between the SHR-G and SHRU-G microbiomes were confined to fewer phyla and classes (Fig. 2).

In addition to variability between stomach and gut phylogenetic composition, the microbiomes varied among the replicate oysters from each site. For some phyla and classes, relative abundances were similar among replicates, and variability (expressed as the standard error of the mean) was similar for each of the three pipelines (see for example Mollicutes and α-Proteobacteria in stomach and gut microbiomes, respectively; Table 1, 2). However, in many cases replicates varied substantially, and the extent of variability differed among pipelines. Mollicutes in the Hackberry Bay gut microbiome, for example, were observed in only 1 of 3 replicates by CloVR and Mothur, and were disproportionately abundant in one replicate according to PANGEA (Table 2).

**Figure 3:**
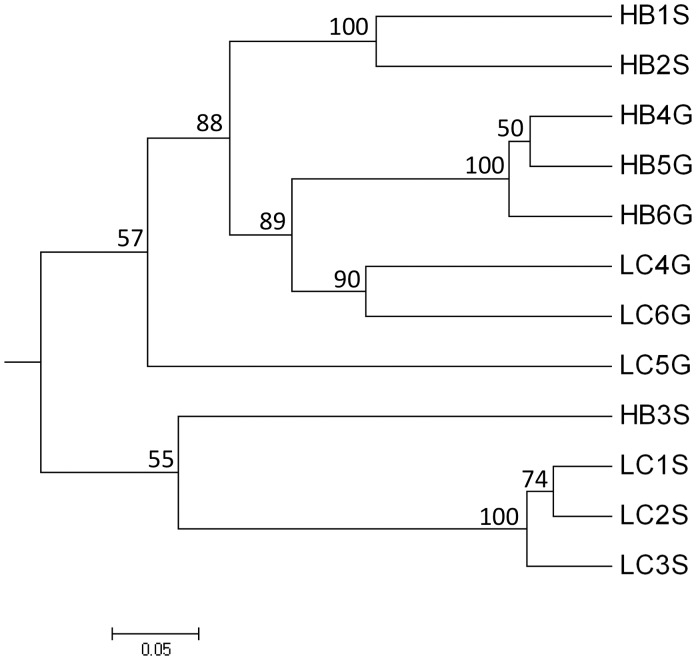
Cluster analysis based on UniFrac distances for sequences derived from the CloVR pipeline for each of the replicate Hackberry Bay (HB) and Lake Caillou (LC) stomach (S) and gut microbiomes (G).

**Figure 4:**
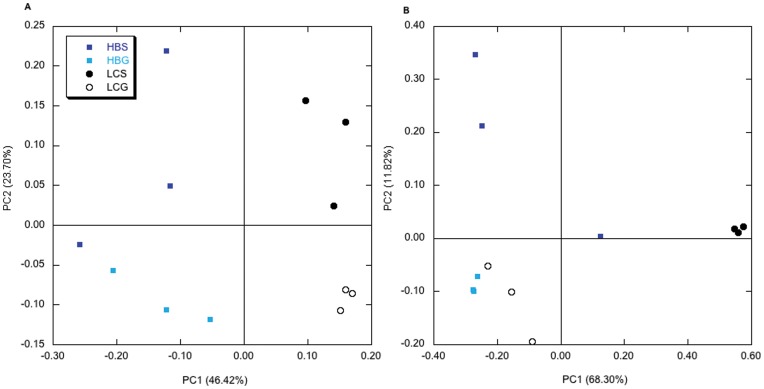
Principal components analysis of unweighted (A) and weighted (B) UniFrac distances for sequences derived from the CloVR pipeline for each of the replicate Hackberry Bay (HB) and Lake Caillou (LC) stomach (S) and gut microbiomes (G).

Variability among replicates was captured by cluster analysis (Fig. 3) and principal components analysis (PCA) of CloVR results using UniFrac distances (Fig. 4a, b), and also by PCA of the relative abundances of classified OTUs (Fig. S1). Results from a cluster analysis using the weighted UniFrac metric showed that the Lake Caillou stomach replicates and Hackberry Bay gut replicates each formed distinct clusters, and that the individual replicates were relatively close in distance. The remaining stomach and gut samples were much less coherent, with replicates resolved at greater distances. Unweighted UniFrac PCA showed that Lake Caillou gut microbiomes clustered together on axis one and two, but that replicates for the other microbiomes were much more dispersed, even though the distinctions between sites and between gut and stomach remained evident (Fig. 4a). Weighted UniFrac PCA, which considered the relative abundances of OTUs, showed that Lake Caillou stomach and Hackberry Bay gut replicates each formed relatively tight clusters on both axes, while replicates for the other microbiomes were dispersed (Fig. 4b). The two stomach microbiomes remained well separated on PCA axis 1, but the gut microbiomes clustered together (Fig. 4b).

## Discussion

We present here the first detailed analyses of *Crassostrea virginica* stomach and gut microbiome compositions. The sample size (triplicate animals for each of two sites) and single sampling time limit extrapolation of the results, but provide a number of new insights. Previous studies have emphasized cultivable members of the gut community, whole animals, pathogens (human and oyster), or specific groups that might contribute to digestion, e.g., *Cristispira* [5], [8]–[11], [13], [25]. Cultivation-free approaches have revealed *Arcobacter* (ε-Proteobacteria) as a major contributor to microbial communities of whole Chilean oysters, *Tiostrea chilensis*, but whole tissue specific associations have not been reported [17]. Hernádez-Zárate and Olmos-Soto [26] have used group-specific FISH and PCR to identify bacteria in *C*. *gigas* tissues, but they did not sequence PCR amplicons or report relative abundances of specific phylogenetic groups. Recently, a PCR and DGGE study of *C*. *virginica* has reported spatial and seasonal differences of whole animal microbiomes for two populations from Maine (USA), but phylogenetic composition has not been assessed qualitatively or quantitatively [27], nor have variations among individual animals been described.

Partial 16S rRNA gene sequences derived from high throughput pyrosequencing as used in this study reveal differences in oyster microbiome composition at several levels, although some of the details of composition vary with the pipeline chosen for sequence analysis (e.g., Tables 1, 2). See Supporting Information S1 for additional discussion of these differences, which do not affect the patterns of variation between stomach and gut microbiomes or variations between sites.

Overall, the results show substantial differences between stomach and gut microbiomes, and between the stomach microbiomes of animals from Hackberry Bay and Lake Caillou (e.g., Fig. 1a, b and 2; Tables 1, 2). In addition, the microbiome compositions of individual replicate animals vary (Fig. 3, 4). Variations between stomach and gut microbiomes likely reflect details of the digestive system, but differences between sites and among replicates suggest that microbiome composition might respond to local factors, and perhaps to genetic differences among individuals. Analogous variability has been reported for other animals [28], [29].

### Oyster Stomach Microbiome

Based on the frequency of OTU occurrence, the stomach microbiome of oysters from Louisiana can exist in at least two states. Mollicutes most closely related to *Mycoplasma* overwhelmingly dominate the classified sequences (>80%) of a state represented by Lake Caillou oysters (Fig. 1a; Tables 1, 3). No other class contributes more than about 2%. Planctomycetes dominate (23%–33%) the alternate state- that of Hackberry Bay oysters (Fig. 1a; Tables 1, 3)- but several other groups also occur in the stomachs of these oysters at modest abundances, e.g., Chloroflexi (8%), Firmicutes (9%–11), Mollicutes (5%–9%), Proteobacteria (5%–12%), and Verrucomicrobia (3%–14%). In addition, two similarly abundant OTUs that belong to different phyla (Planctomyces and either Firmicutes, Tenericutes or Verrucomicrobia) dominate Hackberry Bay oyster stomachs at a species level (evolutionary distance = 0.03; Table 3). The proportion of classified OTUs accounted for by various phyla and classes is also consistent with two distinct states for the stomach microbiome (Fig. 2), although the differences are less pronounced for this metric than for frequency-based estimates of composition. UniFrac PCA (weighted and unweighted) and cluster analyses provide additional support for the “two state” concept (Fig. 3, 4).

The physiological and ecological significance of these oyster stomach microbiomes is uncertain. Dominance by Mollicutes or Planctomycetes is somewhat unusual relative to other microbiomes [28], [30]–[32], although Mollicutes appear abundant in the digestive gland of the Sydney rock oyster (*Saccostrea glomerata*) and in the intestine of the abalone, *Haliotis discus hannai* [33], [34]. Mollicutes have also been reported in oyster gut goblet cells based on microscopic evidence [25], and documented for other invertebrates and fish guts by culture-based and molecular ecological methods [30], [35]–[42]. Otherwise relatively little is known about their associations with invertebrate digestive systems. Indeed, the ecological roles of Mollicutes more generally remain uncertain, with some reports of pathogenesis in selected fish and invertebrates [43]–[46], but other reports indicating some form of commensalism [30], [39].

Thus far, genomic evidence offers few insights, since the genetic repertoire of *Mycoplasma mobile*, the taxon most closely related to the oyster OTUs, is limited in its scope [47]. *M*. *mobile* congenerics in oyster stomachs might simply proliferate using substrate produced by the host or other microbes during digestion; similar suggestions have been made to account for Mollicute associations with cold-water corals [39]. Nonetheless, the possibility that Mollicutes might contribute symbiotically to their hosts cannot be dismissed.

The role of Planctomyces in digestive systems is also uncertain. Although they are ecologically important members of the marine bacterioplankton, functionally diverse and associated with algae, invertebrates and vertebrates [48], [49], they usually occur at relatively low abundances in gut microbiomes (< about 5%) [30]–[32]. However, results from this study suggest that unknown conditions in the Hackberry Bay oyster stomach favor Planctomycete proliferation (Table 1).

It is tempting to speculate here, as others have elsewhere [50], that *Pirellula*-like members of the oyster microbiome exploit sulfated algal polysaccharides for growth, since numerous genes putatively coding for sulfohydrolase enzymes have been observed in the *Rhodopirellula baltica* genome [51], and since sulfated polysaccharides might be commonly ingested by oysters as a consequence of phytoplankton consumption. The ability to use sulfated polysaccharides would thus provide an explanation for Planctomycete abundance. Unfortunately, the phylogenetic relationships between *R*. *baltica* and planctomycete OTUs identified in this study are insufficient to support such inferences. Nonetheless, all *Blastopirellula*, *Pirellula*, and *Rhodopirellula* isolates characterized to date use a wide range of simple non-sulfated sugars [48], [49], [52], at least some of which are likely to occur in the oyster digestive tract as algal biomass is hydrolyzed.

### Oyster Gut Microbiome

The oyster gut microbiome harbors a more speciose or OTU-rich community than does the stomach microbiome based on observed species (S_obs_) and ACE and Chao1 diversity estimators (Table 5). These indices also indicate that stomachs and guts of Lake Caillou oysters harbor fewer OTUs than Hackberry Bay oysters. Thus, OTU richness varies between oyster tissues (e.g., stomach and gut) as has been well documented for the human microbiome [53], but also appears to vary among populations. The source of variations in richness among oyster populations is unknown.

**Table 5:** Diversity indices for Hackberry Bay and Lake Caillou stomach and gut microbiomes at an evolutionary distance D = 0.03.

	Hackberry Bay		Lake Caillou	
Variable	Stomach	Gut	Stomach	Gut
S_obs_	79±10	121±20	18±5	98±18
Ace	148±27	442±140	36±14	252±87
Chao1	232±41	948±315	54±27	411±168
Shannon	3.631±0.222	4.052±0.255	1.269±0.240	3.957±0.268
1/D	24.91±7.92	29.40±7.77	2.32±0.62	35.56±16.47
Evenness	0.40±0.08	0.34±0.05	0.16±0.04	0.49±0.13
Coverage	0.83±0.02	0.68±0.04	0.96±0.01	0.74±0.05

Values are means and standard errors derived from triplicates for each sample, based on analyses by Mothur (excluding singleton and eukaryotic sequences).

Variations in richness notwithstanding, the gut microbiome is not necessarily more diverse than the stomach microbiome based on Shannon and inverse Simpson’s indices and the evenness estimator, each of which are similar for the Lake Caillou gut microbiome and the two Hackberry Bay microbiomes (Table 5). This similarity indicates that in some cases the structure of oyster microbiome diversity (richness and evenness) is independent of the digestive system and phylotype composition. In contrast, all diversity indices for the Lake Caillou stomach microbiome are substantially lower than for Hackberry Bay stomachs, and lower than for both gut microbiomes as well. This can be attributed to the dominance in Lake Caillou oyster stomachs of Mollicute OTUs (e.g., Table 5 and Fig. 1a).

**Figure 5:**
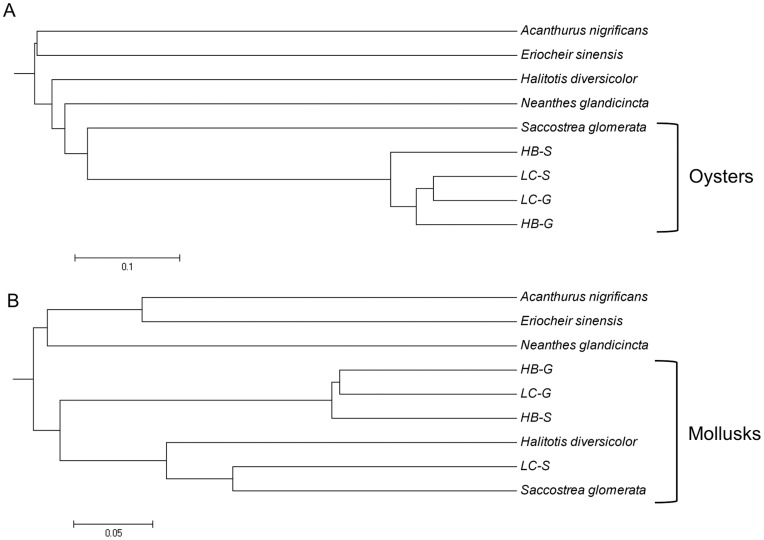
Cluster analysis based on unweighted (A) and weighted (B) UniFrac distances obtained for *C*. *virginica* microbiomes (HB-S, HB-G, LC-S, LC-G as above; this study) and microbiome compositions derived from 16S rRNA gene clone libraries (accession numbers in parenthesis) of an herbivorous coral reef surgeon fish, *Acanthurus nigrificans* (gut sequences FJ653927–FJ65392774 [**68**]); the carnivorous mitten crab, *Eriocheir sinensis* (gut sequences DQ856498–DQ856562 [**69**]); a detritivorous/phytophagus polychaete, *Neanthes glandicincta* (gut sequences FJ618851–FJ618896 [**70**]); the macroalgae-consumng gastropod small abalone, *Haliotis diversicolor* (gut sequences GU070680–GU070693 [**41**]); and the filter-feeding Sydney rock oyster, *Saccostrea glomerata* (digestive gland sequences FM995169–FM995191 [**33**]). All sequences were aligned with the NAST Aligner; a BioNJ tree was used as input for UniFrac. Weighted UniFrac was conducted with normalized sequence abundances. Note that oyster microbiomes form a distinct cluster based on the unweighted analysis (A), while in a weighted analysis (B) all mollusk microbiomes are distinct from crab, fish and polychaete microbiomes; also Lake Caillou oyster stomach microbiomes cluster with the Sydney rock oyster and small abalone microbiomes. Mollicutes dominate all of the latter [Table 1; 33, 41] and account for the observed association.

The composition of gut microbiomes from Louisiana oysters differs from that of other mollusks and from that of other marine and non-marine animals (Fig. 5). Gruenthal [54] has shown that Proteobacteria dominate (>80%) the gut microbiomes of California black (*Haliotis cracherodii*) and white abalone (*H*. *sorenseni*); Actinobacteria, Chloroflexi, Planctomyces and Verrucomicrobia appear to be absent from both. Huang et al. [41] indicate that Mollicutes and δ-Proteobacteria dominate the intestine of the small abalone. Cardoso et al. [55] report that Bacteroidetes and Firmicutes dominate the gut of the gastropod snail, *Achatina fulica*. Firmicutes along with Bacteroidetes, Proteobacteria and Actinobacteria dominate the guts of other invertebrates (e.g., soil-feeding termites [56] and cockroaches [57]) and vertebrates (e.g., herbivorous marine fishes [58]; grass carp, [31], [59] and primates [60]), while Mollicutes dominate the guts of some fish [30], [36]. In contrast, Proteobacteria account for only about 20% of the gut composition of the oysters in this study, Chloroflexi, Planctomyces and Verrucomicrobia are each relatively abundant, and Actinobacteria, Bacteroidetes, Firmicutes, and Mollicutes each contribute about 10% or less (Fig. 1a; Table 3).

These differences in composition among gut systems arise from the effects of multiple interacting variables, including gut architecture, digestive physiology, diet, and the extent to which hosts and microbiomes have evolved symbiotically [61]–[63]. While the effects of some variables, e.g., diet, have clear impacts on some microbiomes [36], [64], [65] the variables that most affect oyster microbiome composition have not been identified. Other than general contributions to heterotrophic metabolism and polymer hydrolysis, the functions of the major members of the oyster gut microbiome are also largely unknown. However, as Mouchet et al. [66] have noted, some functions are likely conserved across microbiomes independent of phylogenetic composition. Thus, the relatively unique composition of the oyster gut microbiome might be functionally similar to the microbiomes of other animal guts.

### Putative Core Microbiomes

Stomachs and guts of oysters in Louisiana harbor a diverse community of bacteria. However, much of this diversity might be due to transient populations that arrive with food sources. During passage through the digestive system OTU abundances might change, in part due to digestion [67], but such changes need not produce or reflect a core community. Although the concept of phylogenetically distinct core microbiomes is controversial [63], to explore its applicability to oysters, we have identified OTUs that are shared among all replicate stomach or gut microbiomes (SHR-S, SHR-G). Note that SHR-S OTUs can occur in some or all of the gut microbiomes, and SHR-G OTUs can occur in some or all stomachs. In addition, we have identified OTUs that are shared among all replicate stomachs or guts and occur uniquely in one type of microbiome or the other (SHRU-S, SHRU-G).

A comparison of SHR and SHRU OTUs reveals that the putative core stomach microbiome consists of only a small fraction of all OTUs (about 2%) representing just three phyla (Firmicutes, Planctomycetes and γ-Proteobacteria). A larger fraction of all OTUs (about 16%) occurs in the putative core gut microbiome, which encompassing 12 phyla and classes (Fig. 2). Mollicutes are notably absent from the putative core microbiome based on SHRU OTUs, which reflects the fact that Mollicutes occur in both the stomach and gut.

The differences between core stomach and gut microbiomes suggest that the stomach might support fewer specific symbiotic interactions, while the gut appears suitable for more phylogenetically and presumably physiologically divergent groups, e.g., Chloroflexi, Crenarchaea, Proteobacteria and Spirochaeta (Fig. 2). Greater niche differentiation in the gut than the stomach might reflect a decrease in bacterial digestion by the host and an increase in the availability and diversity of heterotrophic substrates subsequent to the initial processing of phytoplankton cells in the stomach and diverticula.

The richness of the putative core gut microbiome contrasts with the more limited core microbiomes proposed for zebrafish, an herbivorous bird (the hoatzin, *Opisthocomus hoazin*) and humans [28], [29], [63]. For example, the core zebrafish gut microbiome [28] is comprised of half the number of major phyla and classes that occur in the putative oyster gut microbiome (e.g., 5 versus 10). The core microbiomes of the human gut and hoatzin crop are even more limited, with some arguing that a core gut microbiome for humans might not exist [63]. These observations suggest the possibility that the diversity of core microbiomes might vary systematically among host phyla (e.g. vertebrates and invertebrates), and between terrestrial and aquatic hosts.

### Summary and Conclusions

Relatively deep sequencing of Louisiana oyster stomach and gut contents revealed novel microbiomes that differ from those of other mollusks and other invertebrates and vertebrates. Microbiome composition varied at three levels: between stomach and gut, among replicates at a site, and between sites. These results provide a basis for developing future biogeographically informed analyses based on more extensive temporal and spatial sampling, and comparisons among bivalves and gastropods. Roles for some of the more prominent phylotypes observed, including *Chloroflexi*, *Mollicutes*, *Planctomycetes* and *Spartobacteria* are unknown, but warrant attention, as some of these taxa appear to contribute to a core microbiome that might be conserved within *C*. *virginica* and perhaps other shellfish. Additional effort should also be directed towards understanding the roles of environment variables (e.g., temperature salinity, phytoplankton and bacterioplankton regimes) as factors that shape stomach and gut microbiomes.

## Supporting Information

Figure S1 Principal components analysis of percent composition of each of the replicate Hackberry Bay (HB) and Lake Caillou (LC) stomach (S) and gut microbiomes (G) for sequences derived from the CloVR pipeline; percentages were analyzed using an arcsin transform.
(DOC)

Table S1 Composition of trimmed data sets for three sequence processing pipelines using trim variable values as defined in the text. The percentages of cyanobacterial and eukaryotic sequences reflect removal of singletons and chimeras.
(DOC)

Supporting information S1 (DOC)
