# Supplementary material for: Analysis of Stomach and Gut Microbiomes of the Eastern Oyster (Crassostrea virginica) from Coastal Louisiana, USA
Source: PLoS One. 2012 Dec 12;7(12):e51475. doi: 10.1371/journal.pone.0051475 (PMC3520802; doi:10.1371/journal.pone.0051475)

Figure S1. Principal components analysis of percent composition of each of the replicate Hackberry Bay (HB) and Lake Caillou (LC) stomach (S) and gut microbiomes (G) for sequences derived from the CloVR pipeline; percentages were analyzed using an arcsin transform.


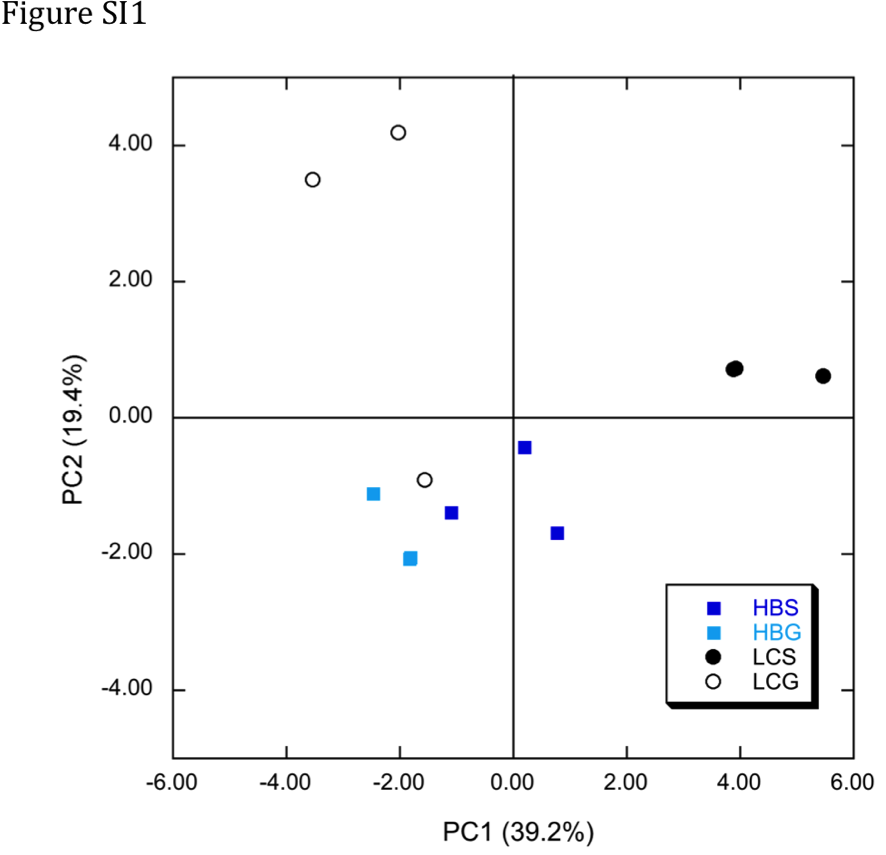

Supplement: Figure S1 — Principal components analysis of percent composition of each of the replicate Hackberry Bay (HB) and Lake Caillou (LC) stomach (S) and gut microbiomes (G) for sequences derived from the CloVR pipeline; percentages were analyzed using an arcsin transform. (DOC) [file pone.0051475.s001.doc]
