# Supplementary material for: Analysis of Stomach and Gut Microbiomes of the Eastern Oyster (Crassostrea virginica) from Coastal Louisiana, USA
Source: PLoS One. 2012 Dec 12;7(12):e51475. doi: 10.1371/journal.pone.0051475 (PMC3520802; doi:10.1371/journal.pone.0051475)
Supplement: Table S1 — Composition of trimmed data sets for three sequence processing pipelines using trim variable values as defined in the text. The percentages of cyanobacterial and eukaryotic sequences reflect removal of singletons and chimeras. (DOC) [file pone.0051475.s002.doc]

Table S1. Composition of trimmed data sets for three sequence processing pipelines using trim variable values as defined in the text. The percentages of cyanobacterial and eukaryotic sequences reflect removal of singletons and chimeras.

| **Variable** | **PANGEA** | **CloVR** | **Mothur** |
| --- | --- | --- | --- |
| **Post-trim sequences** | 199592 | 81486 | 45626 |
| **Singletons** | 11782 | 381 | 719 |
| **Chimeras** | ND | 177 | 71 |
| **% Cyanobacteria + Eukaryote** | 62.7 | 72.7 | 70.7 |
