# Supplementary material for: Analysis of Stomach and Gut Microbiomes of the Eastern Oyster (Crassostrea virginica) from Coastal Louisiana, USA
Source: PLoS One. 2012 Dec 12;7(12):e51475. doi: 10.1371/journal.pone.0051475 (PMC3520802; doi:10.1371/journal.pone.0051475)
Supplement: Supporting information S1 — (DOC) [file pone.0051475.s003.doc]

**Supporting Information**

*Analytical pipeline comparision.*

The post-trim sequence sets produced by PANGEA, CloVR and Mothur differed with respect to the number of sequences retained, the abundance of singletons and the number of chimeras removed. However, similar levels of cyanobacteria and other eukaryotic sequences were observed in each set (Table SI1). Trim variables for Mothur were selected to produce the most stringent screen of the three pipelines. PANGEA and CloVR used default settings and were least and intermediate in stringency, respectively.

Results from CloVR and Mothur pipelines agreed reasonably well for composition and variability among replicates of most major phyla and classes with some exceptions (e.g., Hackberry Bay gut Clostridia). However, greater inconsistencies occurred for some of the minor groups (e.g., Mothur but not CloVR identified Spirochates and Lentisphaerae, while CloVR but not Mothur identified Chlamydiae in Hackberry Bay guts). General agreement between CloVR and Mothur was not surprising since CloVR incorporates part of the Mothur pipeline and uses the same database for sequence classification.

Results from PANGEA were often consistent with those of CloVR and Mothur, but with notable exceptions. PANGEA substantially underestimated members of the Caldilineae in both stomach and gut microbiomes for all sites, and underestimated Planctomyctes in gut microbiomes (see Tables 2 and 3). Relative to CloVR and Mothur, PANGEA also reported a much larger fraction of sequences as unclassified, including 40% and 60% for Hackberry Bay stomach and gut microbiomes, respectively. Some of the differences among PANGEA, CloVR and Mothur can be attributed to the use by PANGEA of a different approach and database for sequence classification than the latter two platforms (Schloss et al., 2009; Giongo et al., 2010; Angiuoli et al., 2011), and to the much larger number of lower quality reads analyzed by PANGEA (Table SI1).

The three pipelines also differ in their ease of use, flexibility and data products. PANGEA is perhaps the easiest to implement, but the least flexible with limited output. Removing specific groups (e.g., eukaryotic sequences) from a data set, which proved necessary for oyster microbiome analyses, must be conducted manually ex post facto. Graphical representations of data and statistical analyses other than paired-sample chi square tests must be created manually from tables of OTU abundances.

CloVR can be implemented readily and produces a rich set of graphical and tabular output. However, the current implementation is also unable to facilitate removal of specific sequence types or phylogenetic groups (e.g., eukaryotes), which necessitates manually recreating graphical and statistical analyses from tabular output of OTU abundance. Mothur is considerably more flexible in its ability to recreate analyses after removing target groups, but requires greater user interaction to produce a complete analysis. For all three pipelines a significant level of manual curation proved necessary to insure that taxonomic identifications of OTUs were accurate. Identifications of Rickettsia were particularly unreliable.

*Composition based PCA analysis of differences between stomach and gut microbiomes*. The UniFrac metric provides a phylogenetically based measure of differences in the membership of stomach and gut microbiomes, and can be incorporated into PCA (and other) analyses. The relative abundance of taxonomic groups identified at specific levels (e.g., phylum or class) can also be used as input for PCA. The results of such an analysis based on results from CloVR (Figure SI1) are generally consistent with those from weighted UniFrac analyses (Figure 4b), but the latter explains a greater percentage of variation among samples (80.1% versus 58.6%). Both analyses show that Lake Caillou stomach microbiomes form a tight cluster distinct from all other samples, and that composition can vary considerably among replicates. However, a composition based analysis indicates that Hackberry Bay stomach and gut microbiomes are less distinct than suggested by a UniFrac based analysis.

*Literature Cited*

Angiuoli SV, et al. (2011) CloVR: A virtual machine for automated and portable sequence analysis from the desktop using cloud computing. BMC Bioinformatics 12:356

Giongo A, et al. (2010) PANGEA: pipeline for analysis of next generation amplicons. ISME J. 4:852-861

Schloss PD, et al. (2009) Introducing mothur: Open-source, platform-independent, community-supported software for describing and comparing microbial communities. Appl. Environ. Microbiol. 75:7537-7541
